# Influence of PBO-FRCM Composite Mesh Anchorage on the Strengthening Effectiveness of Reinforced Concrete Slabs

**DOI:** 10.3390/ma18112583

**Published:** 2025-05-31

**Authors:** Filip Grzymski, Tomasz Trapko, Michał Musiał

**Affiliations:** Faculty of Civil Engineering, Wrocław University of Science and Technology, 27 Wybrzeże Stanisława Wyspiańskiego St., 50-370 Wrocław, Poland; tomasz.trapko@pwr.edu.pl (T.T.); michal.musial@pwr.edu.pl (M.M.)

**Keywords:** FRCM composite, PBO fiber, RC slabs, mesh anchorage

## Abstract

FRCM (Fabric-Reinforced Cementitious Matrix) composites, while providing an effective alternative to FRP (Fiber-Reinforced Polymer) strengthening systems when epoxy resins cannot be used, typically fail to achieve their full strengthening potential. Research indicates that appropriate mesh anchorage systems can minimize some of the undesirable effects that limit FRCM composite performance. This study investigates the effectiveness of different anchorage systems for PBO (p-Phenylene Benzobis Oxazole) fibers in FRCM composites used for strengthening reinforced concrete slabs. A series of unidirectionally bent RC slabs were tested under four-point bending: an unstrengthened control element, slabs strengthened with PBO-FRCM without anchorage, with bar anchorage (GFRP bar in a groove), and with cord anchorage (PBO cord through the slab). The research focused on analyzing the load–deflection behavior and key strain mechanisms that influence structural performance. The findings indicate that a single layer of PBO-FRCM increases bending capacity, raises yield load, and delays initial cracking. Most significantly, the research reveals substantial differences in composite mesh utilization efficiency. This study confirms that mechanical anchorage, particularly bar anchorage, significantly enhances the effectiveness of PBO-FRCM strengthening systems by delaying composite detachment and allowing for greater utilization of the high-strength fiber material. These results contribute valuable insights for RC slabs using FRCM composite systems and the anchorage of their mesh.

## 1. Introduction

Strengthening reinforced concrete structures represents one of the fundamental challenges facing structural engineers today. Years of engineering experience have led to the development of numerous strengthening methods tailored to specific structural elements, enabling the optimization of both construction processes and material usage. Due to the specific behavior and geometry of reinforced concrete slabs, traditional strengthening methods for this type of element most often come down to increasing their thickness. With such strengthening, particular attention should be paid to ensuring the cooperation between the old and new concrete layers of the slab. This approach facilitates the transfer of shear stresses τ at the interface between layers, resulting in greater overall stiffness compared to independently functioning layers of identical total thickness. Such cooperation is achieved through the action of three basic mechanisms: adhesion resulting from chemical and physical bonds between layers, friction between layers resulting from surface roughness, and possibly the “dowel action” effect, which is conditioned by the presence of connectors between layers [1,2]. While traditional slab strengthening methods offer undeniable advantages in terms of simplicity and well-established techniques, they require invasive construction procedures and substantially increase both the dimensions and mass of the strengthened element.

**Figure 1:**
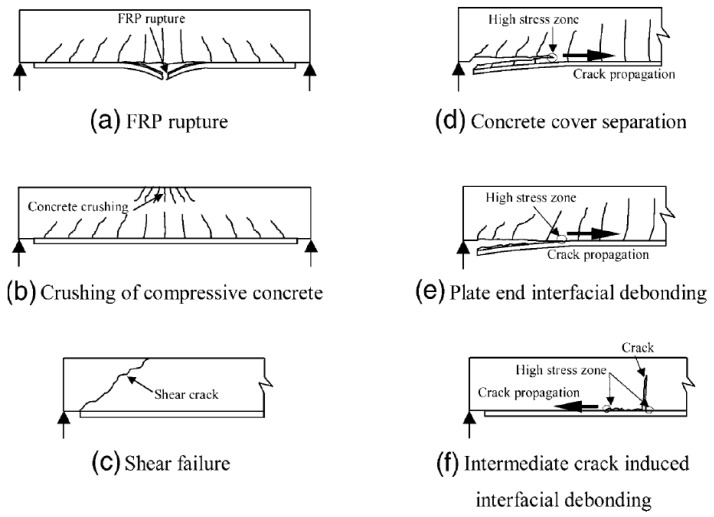
Typical failure mechanisms of elements strengthened in flexure using FRP composites [12].

In order to limit the disadvantages of traditional methods of strengthening building structures, the concept of using composite materials for this purpose was proposed. Most commonly, composites based on polymer matrices (FRP—Fiber-Reinforced Polymers) are used. FRPs have been applied in strengthening structures since the early 1980s [3]. Various types of high-strength fibers are used in these materials, e.g., carbon (CFRP), glass (GFRP), aramid (AFRP), and basalt (BFRP) [4,5]. These composites have confirmed their effectiveness in strengthening different types of reinforced concrete elements such as beams [6,7,8], slabs [9,10], and compressed elements [11]. The high efficiency of FRP composites in strengthening reinforced concrete structures is most often evidenced by the observed failure mechanisms, which are related to the detachment of the composite with the concrete cover layer or the detachment of the composite in the support areas [12]. A graphical representation of the typical failure mechanisms of elements strengthened in flexure with FRP composites is shown in Figure 1.

Pre-tensioning FRP composite strips can substantially enhance their effectiveness by transforming passive strengthening into active strengthening, thereby reducing structural deflections and limiting crack widths under service loads [13]. The main limitation of using FRP composites is their low thermal resistance caused by the properties of epoxy resins, which are characterized by a relatively low glass transition temperature (T_g_ ≈ 50 °C), causing a rapid transition from solid to liquid (viscous) states. However, the conducted research indicates that the degradation of mechanical properties of epoxy resins used in FRP composites can occur even when the temperature threshold of 30 °C is exceeded [14].

**Figure 2:**
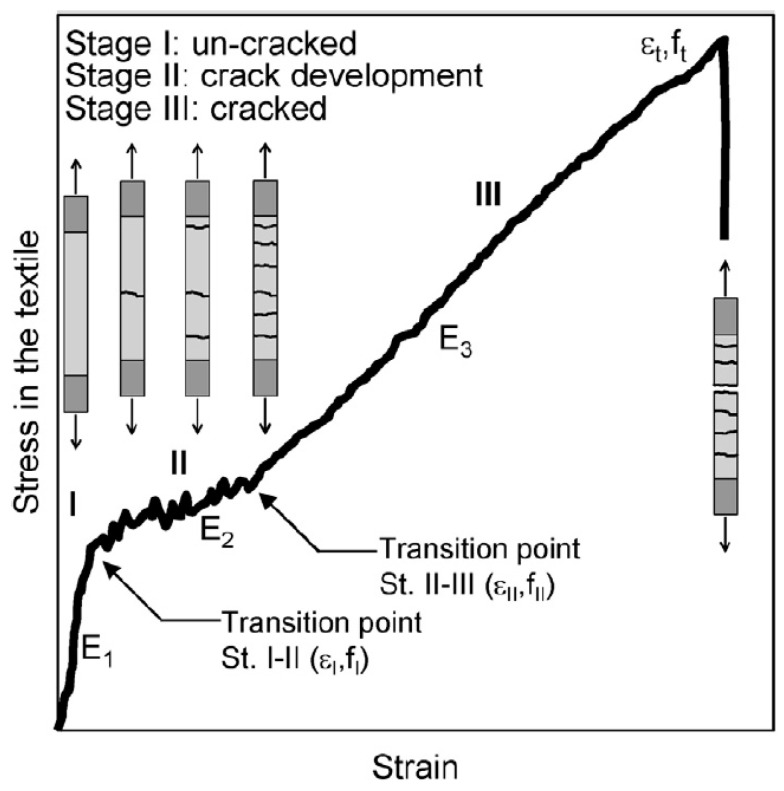
Development of strains during tension of FRCM composite [19].

Since the fibers used in composite materials typically maintain their properties at elevated temperatures, research efforts have strategically focused on developing alternative matrix systems that can withstand thermal exposure. As a result of these considerations, the FRCM (Fabric-Reinforced Cementitious Matrix) strengthening composite was proposed, which also degrades under elevated temperatures [15] but is characterized by significantly greater thermal resistance compared to FRP composites [14]. In FRCM composites, a mineral matrix is used, which, unlike the epoxy resin used in FRP composites, does not lose its properties at elevated temperatures. Due to the significantly lower penetration ability of the mineral matrix compared to the epoxy matrix, in FRCM composites, it was necessary to rearrange the tightly woven fiber mats into meshes with much larger empty spaces between the fabric weaves, which was aimed at enabling the best possible coverage of the fibers with mortar. The theoretical mechanism of FRCM composites under tension is divided into two main phases—the first phase before matrix cracking and the second phase after matrix cracking. The transition between the composite’s load–response phases is defined as the transition point “T”, which is associated with the formation of cracks in the mineral matrix [16,17]. However, in actual tensile tests of FRCM composites, it is indicated that the transition point “T” is essentially an additional intermediate phase of material behavior, which is related to the gradual development of cracking in the matrix, and, therefore, the real behavior of the FRCM composite under tension should be treated as trilinear [18,19,20,21]. The mechanism of strain development in an FRCM composite coupon under tension is schematically shown in Figure 2.

**Figure 3:**
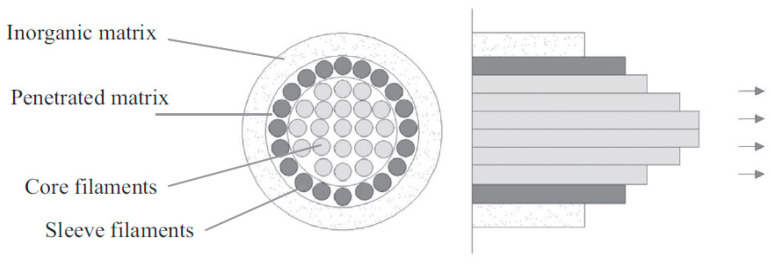
Scheme of the telescopic effect in FRCM composites [22].

Additionally, the actual behavior of the strengthening composite in cooperation with the strengthened structure is influenced by properties related to the adhesion of the composite to the fibers. The low penetrability of the mineral matrix leads to a situation where only the outer layer of fibers from the bundle is well bonded with the matrix. Fibers that do not have direct contact with the matrix can move relative to each other due to reduced friction forces between the fibers, which leads to the telescopic effect [22], resulting in a reduction in the utilization of the fiber potential. The mechanism of the telescopic effect is schematically shown in Figure 3.

An important aspect of the cooperation of the FRCM composite with the strengthened element is also its adhesion mechanisms during debonding. Research indicates that the decisive debonding mechanism occurs at the fiber–matrix interface, not at the composite–concrete interface, which causes the failure mechanisms to be different from those occurring in FRP composites [23,24,25]. This specific behavior of FRCM composites causes the fiber material to be insufficiently utilized, as failure occurs before reaching the full strains of the mesh fibers. In order to reduce the negative impact of mechanisms resulting from the use of a mineral matrix, various modifications of FRCM composites in the form of mesh anchorages are proposed [26,27,28]. Anchorage allows for increasing the effectiveness of mesh utilization from 98–148% [27] to even 235% [28]. In the literature, one can also find effective attempts to increase the effectiveness of FRCM strengthening through substrate grooving, which, in some cases, allows one to achieve full utilization of the composite’s potential [29].

**Figure 4:**
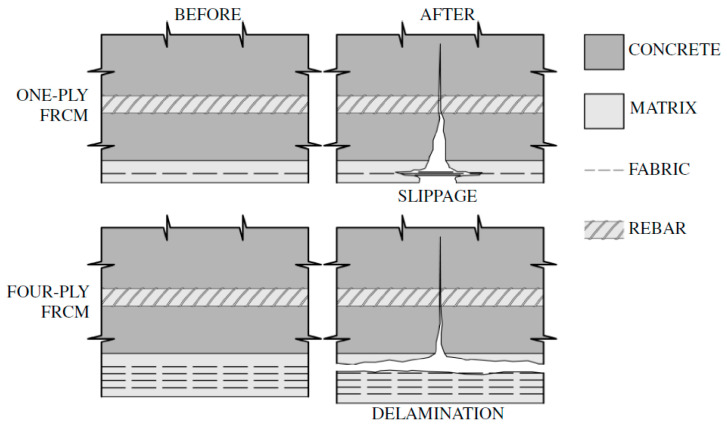
Failure mechanisms of FRCM strengthening in slab elements [31].

The topic of strengthening reinforced concrete slabs with FRCM composites is not widely represented in the literature. Some of the first studies of similar materials on slab elements were described in 2007 [30]. These studies presented carbon fiber meshes embedded in a cementitious matrix, which were compared with traditional FRP laminates. Slippage of the strengthening mesh in the matrix was observed, which caused a reduction in the effectiveness of strengthening. Additionally, a clearly lower stiffness of slabs strengthened with composites based on mineral mortar was observed compared to slabs strengthened with CFRP laminates; however, the ultimate failure forces of the test element were similar. In studies of reinforced concrete slabs strengthened with FRCM composite [31], the influence of concrete strength and the number of FRCM strengthening layers with PBO (p-Phenylene Benzobis Oxazole) fibers on the effectiveness of flexural strengthening was analyzed. Research indicated that strengthening failure occurs due to fiber slippage in the matrix in the case of one layer of composite or detachment of the strengthening from the surface of the strengthened element in the case of four layers of composite, which is schematically shown in Figure 4.

In all analyzed cases, strengthening failure occurred after the reinforcing steel in the strengthened element had yielded. The strengthened slabs carried loads constituting 141% and 205% of the failure force of control samples in the case of lower-strength concrete and 135% and 212% of the failure force of control samples for higher-strength concrete, respectively, for strengthening with one and four layers of PBO-FRCM composite. Along with the increase in the load-bearing capacity of the slabs, a decrease in their ductility was observed, defined as the ratio of the final deflection at failure to the deflection at the yielding of the reinforcing steel. The influence of the slab reinforcement ratio on the effectiveness of strengthening was analyzed in studies of slabs strengthened with FRCM composites with basalt fibers [32]. It was shown that in all slabs with FRCM strengthening, slippage of the strengthening mesh was observed at a load corresponding to about 60% of the failure force; however, the final failure was associated with the rupture of the composite fibers. Slabs strengthened with 1–3 layers of FRCM composite showed increases in load-bearing capacity relative to the unstrengthened element from 11.2% to 98.2%, with the observation that increasing the degree of slab reinforcement reduces the relative increase in load-bearing capacity after strengthening. Comparative studies of FRP and FRCM composite strengthening on slab elements [33,34] showed a decidedly more ductile behavior of FRCM strengthening compared to FRP strengthening, which leads to a greater final deflection at failure. For strengthening with the FRCM system, increases in the load-bearing capacity of the element by 36%, 43%, and 57% were obtained for one, two, and three layers of FRCM composite with PBO mesh, respectively. Additionally, comparative studies were conducted, in which some of the slabs were subjected to environmental factors, which did not show significant changes in the load-bearing capacity of the elements compared to elements maintained in a laboratory environment; however, an approximately 13% increase in the ductility of slabs strengthened with FRCM composite was observed. It was also observed that the strengthening composites attached to the slabs have significantly lower strains than the main reinforcing bars in the slab, which indicates their incomplete utilization. Studies of long-span slabs strengthened with a carbon fiber mesh impregnated with epoxy resin and embedded in a mineral matrix indicated increases in the load-bearing capacity of the element, from 67% for one layer of mesh up to 245% for four layers of mesh [35]. The application of strengthening and increasing the number of its layers positively affects stiffness, which leads to obtaining smaller deflections at given load values. In addition to increasing the load-bearing capacity and stiffness, strengthening based on carbon fiber in a mineral matrix leads to obtaining a more favorable cracking pattern of the element—a larger number of smaller cracks. In studies of small, unidirectionally bent slabs strengthened in flexure with PBO-FRCM composite, the strains of the composite in the main bending direction and in the direction perpendicular to it were determined [36]. The obtained level of strengthening was over 250% for two layers of PBO-FRCM strengthening, and the strains of the composite in the direction perpendicular to the bending direction were about 1/4 of the main longitudinal strains, which is a significant value and may be important in terms of force redistribution in the element. In studies of multidirectionally bent slabs strengthened with FRCM composites, different composite arrangements were compared to optimize their use [37]. The strengthening failed due to fiber slippage inside the matrix or partial rupture of the used carbon and glass fibers. All types of strengthening applied contributed to significant increases in the load-bearing capacity and stiffness of the tested slabs; however, the most effective method of strengthening was considered to be the classic distribution of the composite over the entire surface of the slab. Additionally, the influence of pre-cracking of the slab, before strengthening with FRCM composite, was tested, which was supposed to simulate the real behavior of the structure. This caused a reduction in the effectiveness of strengthening in the initial phase of loading; however, at the moment of full mobilization of the composite, the influence of pre-cracking was not significant, as the slab behaved almost identically to the others. In similar studies concerning multidirectionally bent reinforced concrete slabs strengthened with FRCM composite with carbon fibers, attempts were made to calibrate numerical models for parametric analysis used to optimize the strengthening layout [38]. In the studies, an increase in element load-bearing capacity ranging from 115 to 206% was achieved depending on the number of layers and strengthening arrangement. The conducted parametric analysis indicated that the developed numerical model has very good convergence with the experimental results, and, based on this, recommendations were provided for the optimal distribution of strengthening depending on element dimensions. Slabs strengthened with FRCM composites were also tested for the punching problem characteristic of this element [39]. It was determined that the influence of this type of strengthening on the punching resistance is relatively small and, with two layers of carbon fiber mesh, ranges from 9.1% to 18.1% depending on the concrete strength used. It was observed that the application of strengthening also causes an increase in the amount of energy absorbed during the failure of the element, which improves the properties of the residual load-bearing capacity of elements after punching. In studies of the influence of composites based on mineral mortar used to strengthen slabs damaged under fire conditions [40], basalt fiber meshes were used, which were impregnated with epoxy resin and then bonded to the structure by shotcreting. The applied strengthening allowed for recovering the original load-bearing capacity of the elements, even exceeding it by a value from 31% to 127%. In slab elements, studies of mineral matrix modifications of composites were also proposed, which are supposed to improve their properties. The addition of synthetic fibers has a small effect on load-related cracking and the peak load-bearing capacity of the element; however, as a result of the application of additional synthetic fibers, cracking related to the shrinkage of the mineral matrix was significantly reduced [41].

The authors of this study did not find any descriptions in the anchorage of FRCM composites in slab elements. Studies of anchorages of composites strengthening reinforced concrete slabs were previously conducted only with the use of FRP composites [9,10]. The effectiveness of strengthening after anchoring the composite increased by 30% [9] or even by 44% [10] compared to strengthening without anchorage. At the same time, it was observed that the failure of elements was associated with the failure of the anchorage, which further indicates its effectiveness. The lack of research related to the anchorage of FRCM composites in slab elements, the effectiveness of anchorages in other elements strengthened with FRCM composites, and the effectiveness of anchorages in slab elements strengthened with FRP composites prompted the authors to take up this topic and conduct research and analysis related to it. In most of the cited studies, similar schemes of FRCM composite failure are repeated, which cause ineffective utilization of fibers, especially PBO fibers, which almost never rupture; therefore, it is important to undertake the research attempts carried out in this study. The main goals of this work were to determine the effect of the proposed anchorage on the load-bearing capacity and deformability of reinforced concrete slabs strengthened with PBO-FRCM composite, to evaluate the influence of strengthening and its anchorage on the development of cracking in the element and to determine the effectiveness of the proposed anchorages. To achieve the goals of this work, a series of destructive tests were prepared on reinforced concrete slabs strengthened with PBO-FRCM composite using different types of anchorage.

## 2. Materials and Methods

**Figure 5:**
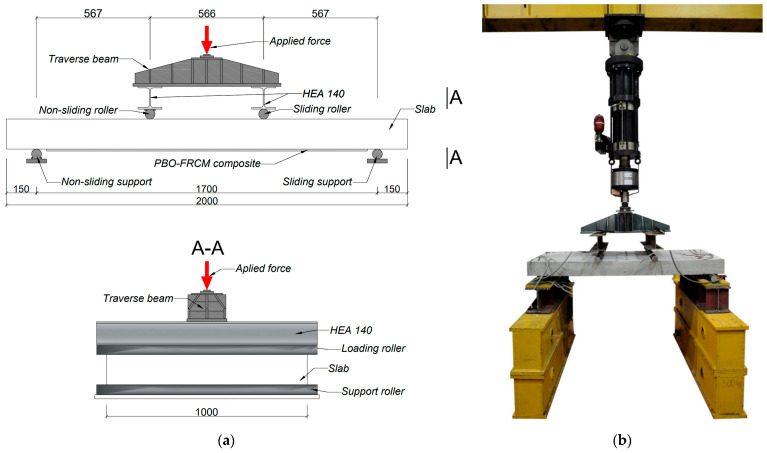
Test stand used in the research: (**a**) scheme with dimensions (in millimeters), (**b**) photo of real test stand used in the research.

The experimental program employed seven unidirectionally bent reinforced concrete slabs measuring 150 mm in thickness, 1000 mm in width, and 2000 mm in length. The slabs were positioned on linear hinged supports located 150 mm from each edge, creating an effective span length of 1700 mm. Testing followed a 4-point bending configuration with loading points positioned at one-third of the span length (~566 mm apart). The linear application of force was ensured by steel rollers, which were additionally stiffened from above with steel I-beams attached to the traverse beam, through which the force was transmitted from the hydraulic actuator. The scheme of the test stand is shown in Figure 5.

The main bottom reinforcement of the slabs consisted of 8 bars with a diameter of 10 mm (spacing of about 125 mm), and 8 bars with a diameter of 8 mm were used as the top reinforcement (spacing of about 125 mm). In both the upper and lower reinforcement mesh, secondary reinforcement, perpendicular to the main reinforcement, was applied, which was made of bars with a diameter of 8 mm at a spacing of 200 mm. The strength parameters of the reinforcing steel and concrete are presented in Table 1.

**Figure 6:**
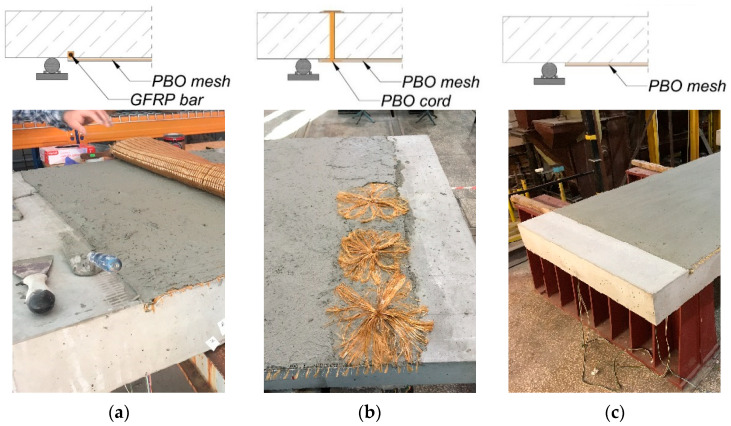
FRCM composite support zone geometries used in the research: (**a**) bar anchorage—type A, (**b**) cord anchorage—type B, (**c**) no anchorage—type C.

The reinforced concrete slabs were made in a prefabrication plant and then left to cure for 28 days. One of the slabs was designated as a control element; therefore, it was not subjected to any additional treatments. The remaining 6 slabs were strengthened in flexure with FRCM composite with PBO mesh. The slabs were strengthened by attaching one layer of composite to the bottom surface, spread over the entire width of the slab and over a length of 1600 mm (the edge of the strengthening composite was located 200 mm from the end edge of the slab and 50 mm from the support during the test). Among the strengthened slabs, different types of composite support zone geometries were applied, which are schematically shown in Figure 6. Two strengthened slabs were left without any anchorage of the composite (type C); in two slabs, the composite was anchored by winding its mesh on a GFRP bar, which was glued into a previously prepared groove (type A); and in the remaining two strengthened slabs, the composite was anchored by threading a PBO cord through the slab and connecting it with the composite mesh through radial distribution of the cord fibers (type B). The used test elements are summarized in Table 2.

The test elements were loaded using Instron (Norwood, MA, USA) hydraulic actuator with a maximum loading force of 500 kN controlled by displacement. Instrumentation included 50 mm electrical resistance strain gauges paired with Hottinger Baldwin Messtechnik MGCPlus (Darmstadt, Germany) data acquisition system to monitor longitudinal strains in the compressed concrete zone, tensioned concrete surface, and strengthening composite mesh. Additional 20 mm strain gauges measured longitudinal strains in the main reinforcing bars throughout loading. Additionally, the slab deflections and vertical displacements above the supports were monitored, for which LVDTs (Linear Variable Differential Transformers) with an accuracy of 0.001 mm paired with Hottinger Baldwin Messtechnik MGCPlus data acquisition system were used. In selected elements, fiber optic (DFOS—Distributed Fiber Optic Sensing) measurement of deformation of the tensile surface of concrete and the strengthening composite was also used, conducted using LUNA OBR 4600 (Roanoke, VA, USA) optical backscatter reflectometer.

## 3. Results

### 3.1. Load–Deflection Relationship and Failure Mechanisms

**Figure 7:**
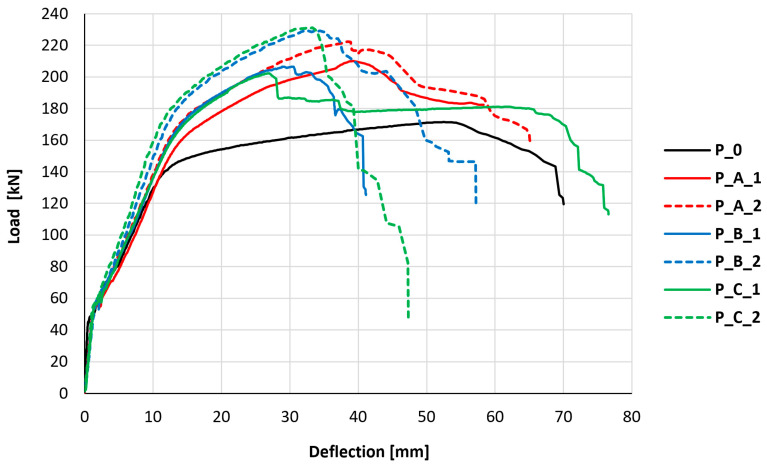
Load–deflection relationship for all tested elements.

The primary observed issue was the influence of PBO-FRCM strengthening and its mesh anchorage on the load–deflection relationship. The element’s deflection was considered to be the reading from the LVDT located in the middle of the slab span, between the places of force application. The results of the deflection values correlated with the readings from the force gauge in the hydraulic actuator are presented in the graphs shown in Figure 7.

**Figure 8:**
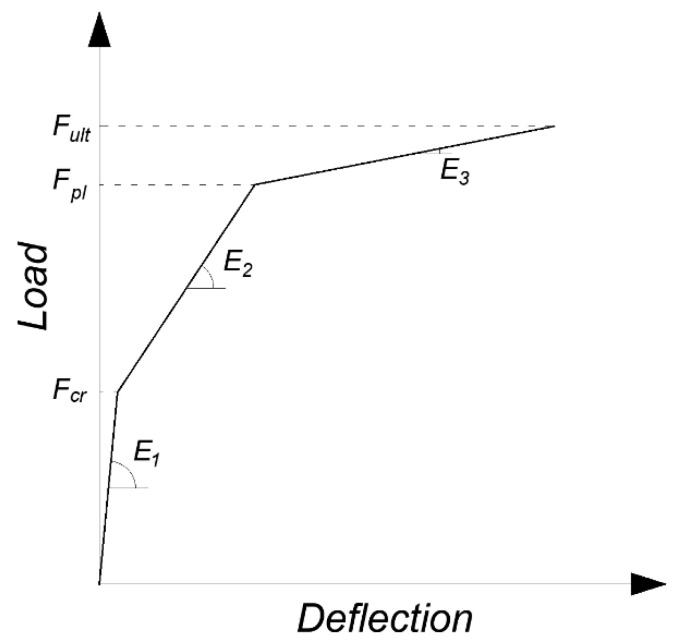
Schematic trilinear relationship of slab behavior along with descriptions of basic mechanical properties.

All experimental results demonstrate a consistent three-phase behavior pattern across the tested slab elements. The first phase begins upon initial loading and continues until the onset of cracking. Following cracking, the load–deflection curves exhibit a reduced slope, characterizing the second phase that continues until the main reinforcing bars yield. The next, third deformation phase begins immediately after the yielding of the tensile reinforcement and is characterized by an even smaller slope of the curve than observed in the second phase. The third phase ends with the failure of the slab and the loss of ability to carry higher loads. To facilitate systematic comparison across specimens, a trilinear theoretical model was developed that characterized the behavior through key transition points and distinct slope changes corresponding to each performance phase. The used notations and the described comparative model are shown in Figure 8, and Table 3 summarizes the most important parameters described by the model. The value of the curve slope in the first phase of the element’s behavior was omitted in the summary, due to the low deflection values in this range and the large influence of relative measurement error.

The behavioral differences between strengthened and non-strengthened elements become pronounced during the second and third phases (post-cracking stages). The difference in the stiffness of elements in the second phase is not significant, as, depending on the element, it is 3–33%; however, in the third phase, the stiffness of strengthened elements is 262–353% greater compared to the element not strengthened with the PBO-FRCM composite. This indicates that strengthening begins to provide benefits only after the element has cracked and shows the greatest efficiency in terms of increasing stiffness after the main reinforcement has yielded. Differences in the behavior of slabs were also observed in terms of the loads at which the transition between individual phases of the slabs’ load response occurs. Already at the first change, associated with the moment of cracking, the evident influence of PBO-FRCM strengthening is noticeable. Strengthened elements cracked at loads 18–26% higher than the control element. A similar increase in force at the phase change was observed in the case of yielding of the main reinforcement, which, in strengthened elements, occurred at loads 17–30% higher than in the control element. The maximum load on the slabs for strengthened elements was 18–35% higher than in the control element. These consistent increases in force capacity across characteristic phases indicate that a single layer of PBO-FRCM composite provides an approximately 27% improvement in the strengthening effectiveness of the tested reinforced concrete slabs. The variability across individual test results prevents the precise quantification of how composite anchorage directly influences the load-bearing capacity and stiffness during specific mechanical response phases. Thus, the conclusion is that anchorage does not significantly impact these particular performance metrics.

In each element tested, the failure mechanism of the slab was appropriate for this type of element and resulted from the loss of load-bearing capacity through yielding of the main (steel) reinforcement. In the case of strengthened elements, the exhaustion of the load-bearing capacity of the steel reinforcement was preceded by the exhaustion of the load-bearing capacity of the PBO-FRCM composite strengthening. This caused the transfer of forces (previously carried by the strengthening) to the base element (including its reinforcement). The failure of strengthened elements has a somewhat more brittle character (higher load at smaller deflection) compared to the reinforced concrete element without strengthening, which is manifested by a much more sudden drop in load-bearing capacity after reaching the maximum loading force.

**Figure 9:**
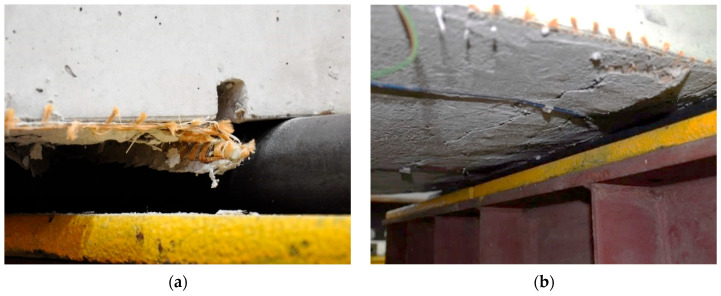
Failure mechanism of bar anchorage: (**a**) type A, (**b**) type B.

For specimens with anchored strengthening systems (types A and B), anchorage failure consistently preceded composite failure, highlighting the critical role of these connection mechanisms in the overall system performance. In the case of bar anchorage (type A), the failure of the anchorage proceeded through a sudden pullout of the anchoring bar from its groove. The sudden loss of load-bearing capacity in elements strengthened with bar anchorage of the composite mesh could also result from the loss of restraint of the mesh ends, which caused a tendon-like behavior in the strengthening. In the case of cord anchorage (type B), the character of the anchorage failure was indistinct and more ductile, as no sudden rupture of the cord was observed but a gradual slippage of fibers and slow pulling of the cord from the vertical hole. This also indicates that the cord anchorage of the composite mesh is not able to create a tendon-like behavior in the strengthening, which can be observed in the case of strengthening with bar anchorage. The typical failure mechanisms of anchorages for types A and B are shown in Figure 9. Important from the safety point of view of strengthening is also the maintenance of the partial post-critical load-bearing capacity of elements with anchored strengthening. Both elements strengthened with bar anchorage (type A), despite exceeding the point of maximum load, did not completely lose their load-bearing capacity and indicated a relatively high level of residual load-bearing capacity.

### 3.2. Cracking Development

**Figure 10:**
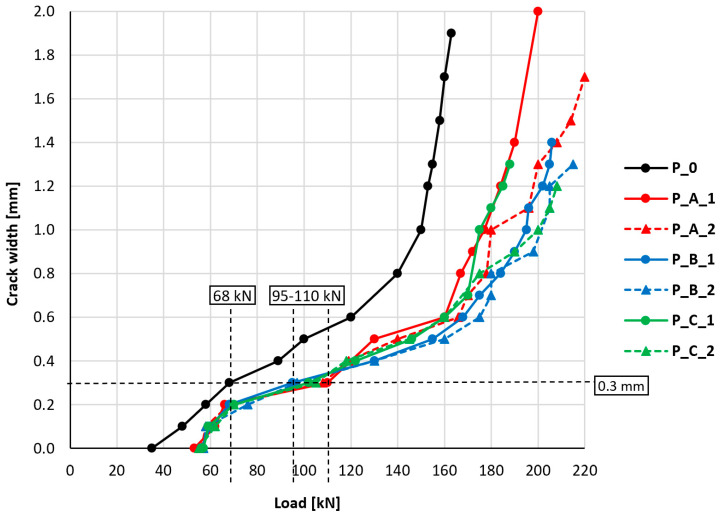
Relationship between crack width and slab load.

During the tests, the crack width on the side surface of the slab was also observed. The measurements were made using a magnifying glass with a scale. The graph shown in Figure 10 indicates the relationship between the maximum observed crack width and the slab load. A crack width of 0.0 mm was considered to be the moment of the first observed cracking visible on the side surface of the slab. Table 4 summarizes the key values related to the cracking of reinforced concrete elements—the moment of the first crack formation and the moment of exceeding the limit values of crack width in reference to the Serviceability Limit State according to Eurocode 2 [42], which are 0.3 and 0.4 mm.

In the course of the relationship between crack width and slab load, a different character of the unstrengthened slab’s behavior was distinguishable compared to the strengthened ones. Cracking of strengthened slabs became apparent at loads 51–63% higher compared to the unstrengthened slab. Achieving the lower level of crack width limitation imposed by Eurocode 2 (0.3 mm) in strengthened slabs occurred at loads 40 to 62% higher than was the case for the unstrengthened slab. The analogous differences for the higher level of crack width limitation (0.4 mm) ranged between 33 and 46%. The results of the research in this area indicate the positive influence of PBO-FRCM strengthening on delaying the moment of crack formation and limiting the width of cracking in the tested elements. The differences between the behavior of individual strengthened slabs with different types of anchorages are slight and were considered insignificant.

The high variation in measurement results in the later stage of loading the slabs does not allow for drawing accurate quantitative conclusions. However, a clear tendency is observed, which allows for drawing an unambiguous conclusion about the positive influence of PBO-FRCM composite strengthening on the cracking resistance of the structure and the limitation of crack width with further increases in the load. The differences between the behavior of individual strengthened slabs with different types of anchorages are, however, slight and were considered insignificant, especially in the typical design range of reinforced concrete elements.

**Figure 11:**
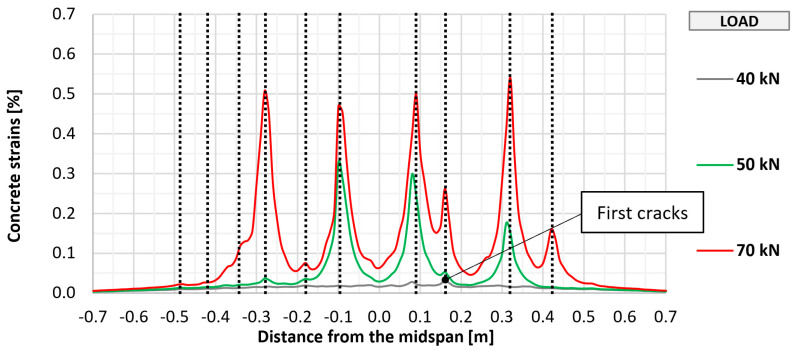
Distribution of longitudinal strains of the bottom concrete surface of slab P_0.

**Figure 12:**
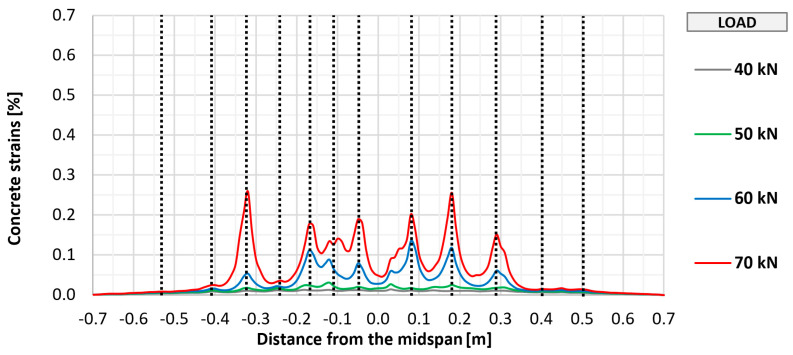
Distribution of longitudinal strains of the bottom concrete surface of slab P_A_2.

**Figure 13:**
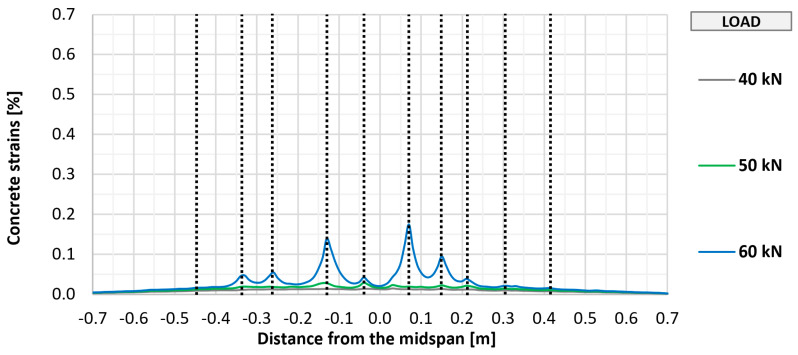
Distribution of longitudinal strains of the bottom concrete surface of slab P_B_2.

**Figure 14:**
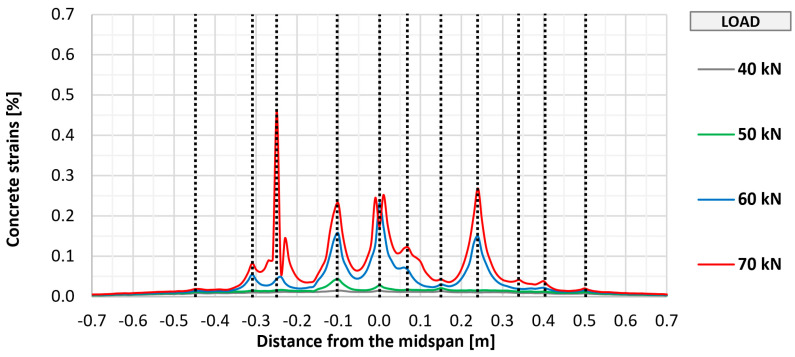
Distribution of longitudinal strains of the bottom concrete surface of slab P_C_2.

The cracking of the bottom concrete surface in selected elements (P_0, P_A_2, P_B_2, and P_C_2) was also monitored using fiber optic sensors, which made it possible to perform a geometrically continuous measurement, which was important due to the possibility of indicating the place of crack formation. The measurement results along with the marked places of crack formation are shown in Figure 11, Figure 12, Figure 13 and Figure 14. The vertical dashed lines in the Figures indicate crack locations.

Similar to the visual assessment, significant differences were observed between strengthened and unstrengthened elements. The measurement with the recorded first cracking occurred at a higher load in strengthened elements (about 50 kN) than in unstrengthened elements (about 40 kN). In each tested element, a similar number of perpendicular cracks formed, ranging from 10 to 12. In all cases, the cracking had a similar uniform distribution, with the greatest concentration in the area of constant bending moment between the points of load application. Further increasing the loads caused a proportional development of cracking with a significant limitation of strains in strengthened elements compared to the unstrengthened element. No significant differences were observed between the different types of anchorage in terms of the development of cracking in the strengthened element.

### 3.3. Steel Reinforcement Strains

**Figure 15:**
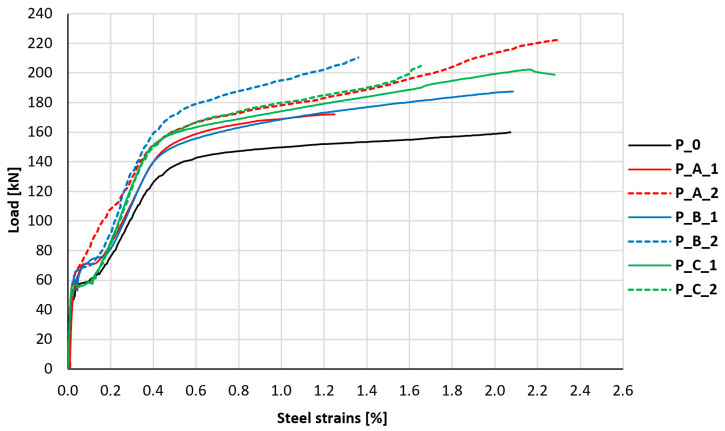
Graph of the relationship between the strains of the main reinforcement bars and the load.

Another important parameter related to the load-bearing capacity of reinforced concrete slabs is the stress level of their main tensile reinforcement. During the loading of the test elements, the strain of the bottom reinforcement bars was monitored in all slabs. Representative results of the strain of the middle bottom reinforcement bars in the middle of the slab span are shown in Figure 15.

All load–strain relationships presented in the graph are characterized by a similar course, with differences in load values at individual strain levels. The first break in the graph, which is associated with the cracking of the element, appears much more quickly in the case of the unstrengthened control element. It occurs at a load level of about 55 kN, while in the remaining slabs, it occurs only at a level of about 67 kN, which is associated with an increase in the stiffness of the uncracked cross-section and partial assumption of loads by the composite layer. Further loading of the unstrengthened element causes significantly higher strains of the main reinforcing bars compared to the strengthened elements. A similar early first break in the graph (approx. 55 kN) is visible in both elements strengthened without anchorage (P_C_1 and P_C_2); however, with further loading, the character of the reinforcement behavior of these slabs was more similar to other strengthened slabs. The breaking of the graphs for slabs P_C_1 and P_C_2 at lower loads compared to other strengthened elements, and the unusual graph of reinforcement strains for element P_A_2 (in which the typical breaking of the graph in the initial phase of cracking development is not noticeable), was most likely caused by random factors, resulting from the random distribution of cracks in the element. The random distance of the measurement location relative to the location of the crack had a significant impact on the values of the read strains directly after the element cracked. The reduction in strains of the main tensile reinforcement at a similar level of loads is a very clear indicator of the effectiveness of the applied strengthening.

For comparative purposes, a reference level of the main reinforcement strains equal to 1% was adopted, for which the recorded load values in strengthened elements are, on average, 18% higher compared to the unstrengthened element. In the case of elements strengthened without anchorage, the levels of these loads are, on average, 18% higher, for elements with bar anchorage of the composite mesh 16% higher, and for elements with cord anchorage of the composite mesh, on average, 21% higher. This indicates the slightly higher effectiveness of PBO-FRCM composites with cord anchorage (type B) in relieving the main reinforcement compared to elements strengthened without anchorage or with bar anchorage (types C and A); however, the differences between these values are small.

### 3.4. Concrete Strains

**Figure 16:**
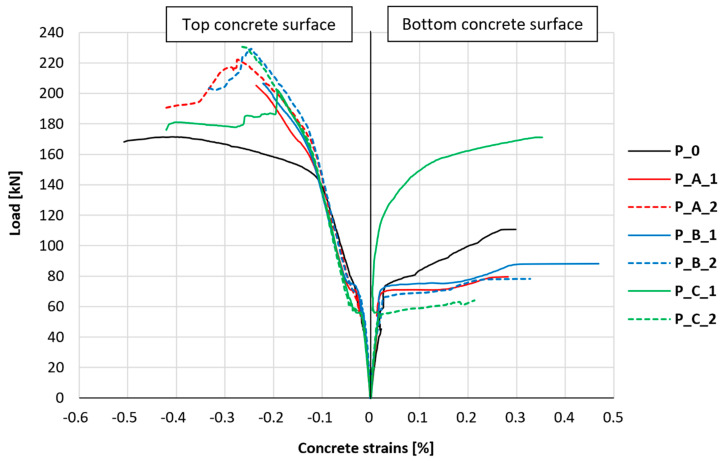
Graph of the relationship between the strains of the top and bottom concrete surfaces and the load.

The longitudinal strains of the bottom and top concrete surfaces in the middle of the slab span were also monitored. The results of measurements of the strain of compressed concrete (top surface of the slab) and tensile concrete (bottom surface of the slab) in the middle cross-section of the slab and in its middle longitudinal axis for all research series are collectively presented in Figure 16. The graph and analysis omitted data related to the tensile concrete of the test element P_A_2 due to damage to the strain gauge in the early phase of cracking.

In the case of tensile concrete, an equal development of strains can be observed in all elements, both the unstrengthened element and the strengthened elements. This tendency is maintained until the moment of element cracking. After the element cracks, depending on the exact position of the crack, each of the tested elements shows different behavior; however, in most cases, it should be considered that the through-going crack led to damage to the strain gauges, and comparing further measurements was considered unreliable. More stable results were obtained for the compressed concrete surface. Similar to the case of tensile concrete, the strains of compressed concrete are almost identical for all tested elements at the beginning of the loading process. After cracking, the strains of compressed concrete also take very similar values for all tested elements, and differences begin to be noticeable at loads at which the tensile reinforcement of the slab yields. Furthermore, the slope angle of the graph of the relationship between longitudinal strains of compressed concrete and load is greater in the case of strengthened elements and is similar for each strengthened element, regardless of the type of anchorage applied. Table 5 summarizes the loads at which the breaking of the graphs described above occurs and the slope of the of the graphs in the last phase of the slabs’ deformation.

The change in the slope of the graph of longitudinal strains for compressed concrete in strengthened elements occurs at a load 9–21% higher than in the case of the unstrengthened element. The slope of the graphs, with further development of longitudinal strains of compressed concrete after the phase associated with the yielding of tensile reinforcement in the case of strengthened elements, is approximately 4–5-times greater for strengthened elements than for the unstrengthened element. This indicates that in the last phase of the slab’s load–response behavior, PBO-FRCM strengthening also affects the stress level of compressed concrete in the element. However, no clear tendency was observed that could indicate differences in this aspect due to the type of composite mesh anchorage applied. Additionally, attention should be paid to the somewhat more plastic character of the transition between the described phases of compressed concrete behavior in the case of strengthened elements, which manifests itself in a much more elongated transitional phase, which can be noticed in the form of an arc between the two linear phases of deformation.

### 3.5. Strengthening Composite Strains

**Figure 17:**
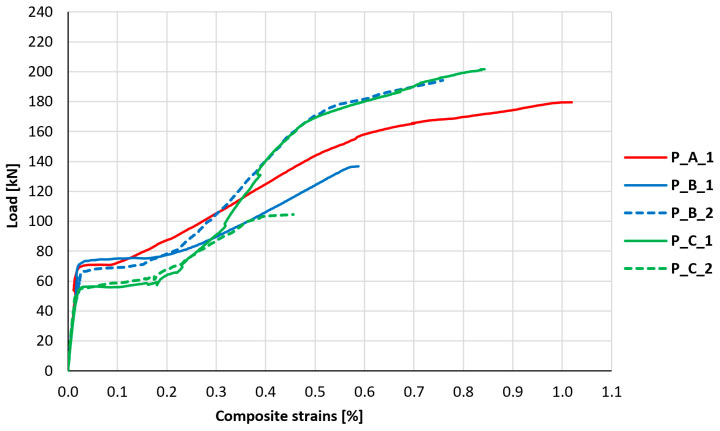
Graph of the relationship between composite strains and load.

The most important parameter from the point of view of assessing the behavior of the strengthening composite, and its mesh anchorage, is the measurement and analysis of its strains under the influence of element loading and relating these strains to the limit value for the used fibers. The results of measurements of longitudinal strains of the composite in the middle of the slab span, in its middle axis, are shown in Figure 17. The relationship obtained in measurements made in element P_A_2 differed significantly from the values obtained in the other research series, which suggests a failure of the strain gauge; therefore, these measurements were rejected from further analysis. The control element P_0 was omitted in the analysis, due to the lack of PBO-FRCM strengthening.

In the initial phase of development of tensile strains, all obtained graphs were almost collinear, and no significant differences were observed between them. The phase of initial strain increase is interrupted by a stage of sudden strain increase, which exhibits itself as a horizontal section on the graph, which appears at different load levels depending on the element. Further development of strains manifests itself on the graph in the form of a curvilinear section until the strain gauge is damaged.

Table 6 summarizes the characteristic values associated with the relationship between longitudinal strains of the composite and the load—the load associated with the start of the stage of sudden strain increase, the maximum recorded strain of the composite, and the load associated with it. Additionally, the table compares the maximum recorded strains to the maximum strains of the PBO mesh at failure (*ε*_frcm,ult_ = 2.15%) and the load associated with the maximum strain of the composite to the tested load-bearing capacity of the element. Based on the values above, an auxiliary mesh utilization indicator was determined, being the quotient of the ratio of the maximum recorded mesh strain to the failure strains and the ratio of the maximum recorded load to the load-bearing capacity of the slab to the load at which the last measurement was recorded. The mesh utilization indicator was calculated based on Formula (1).

(1) $$I_{frcm,max}=\frac{\varepsilon_{frcm,max}/\varepsilon_{frcm,ult}}{F_{frcm,max}/F_{ult}}$$

The largest strains of FRCM strengthening, corresponding to almost 50% utilization of its potential, were recorded on element P_A_1, in which the mesh was anchored using a GFRP bar. In the remaining elements, strains corresponding to only 20–40% of the potential of PBO-FRCM strengthening were recorded. However, it should be noted that the end of the strain reading from the strain gauge attached to the strengthening did not always coincide with the failure of the element and very often occurred much earlier. The corrected values of strengthening utilization, expressed by means of the mesh utilization indicator, indicate that, in the case of type A anchorage, there was a potential for 55% utilization of strengthening; in the case of type B anchorage, it was 41% and 42%; and in the case of no anchorage (type C), 39% and 47%. Among all the tested elements, based on the conducted analysis, it is noticeable that the increase in potential through cord anchorage (type B) is not significant, and in the case of bar anchorage (type A), there is a notable increase in the utilization of strengthening potential. Assuming an average value of the utilization indicator for elements of series C equal to about 0.43, the increase in the utilization indicator through bar anchorage (type A) to a value of 0.55 is associated with an approximately 28% increase in strengthening effectiveness.

**Figure 18:**
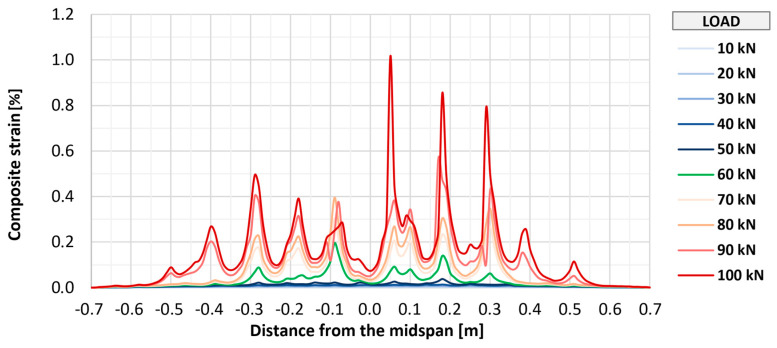
Distribution of longitudinal strains of strengthening of slab P_A_2.

**Figure 19:**
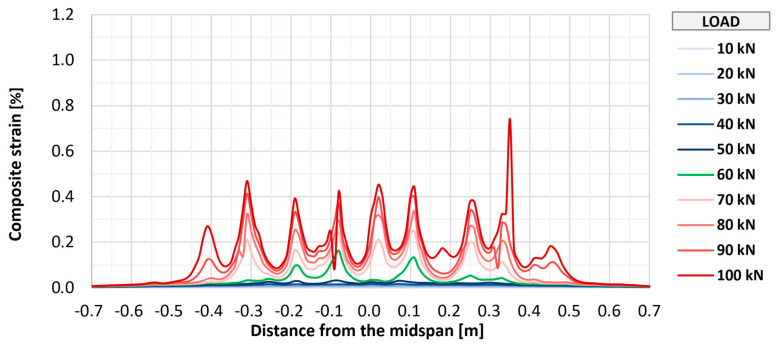
Distribution of longitudinal strains of strengthening of slab P_B_2.

**Figure 20:**
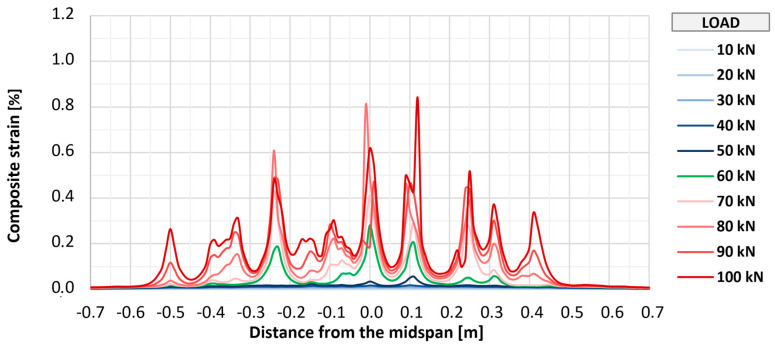
Distribution of longitudinal strains of strengthening of slab P_C_2.

Fiber optic measurements applied on three of the strengthened elements (P_A_2, P_B_2, and P_C_2) allowed for monitoring the strains of the composite over its entire length. Figure 18, Figure 19 and Figure 20 show the distributions of longitudinal strains of strengthening for individual elements in the longitudinal axis of the slab in the range of loads associated with the initial development of strengthening strains (up to 100 kN). Further phases were omitted from the graphs to improve their readability and due to large measurement errors resulting from the dynamic development of cracking. Shades of blue indicate the early phase of strain development, in which the composite works in an almost uncracked state. The transitional phase, in which the first clear peaks indicating cracking appear, is marked in green, and the phase of intensive development of cracking is marked in shades of red.

In all elements, regardless of the type of anchorage, the initial development of composite cracking was observed at a load value of 60 kN. At this stage, in all tested elements, the maximum strains were at a similar level from about 0.15% to 0.25%, and in each element, about seven to nine peaks suggesting the location of cracking were observed. With further increases in loads to a level of 100 kN, an increase in maximum strains in each element to a level of about 0.7% to 0.8% was observed, as well as about 9–10 main peaks indicating the location of crack formation. At this level of loads, no significant differences were observed between the individual research series, and any deviations between series were caused by the random nature of structural cracking. The number of cracks observed along the length of the strengthening is similar to that determined for the bottom surface of concrete, which suggests that the FRCM strengthening takes the greatest strains at the place where a crack forms on the concrete structure.

### 3.6. Strain Distribution Within the Element

**Figure 21:**
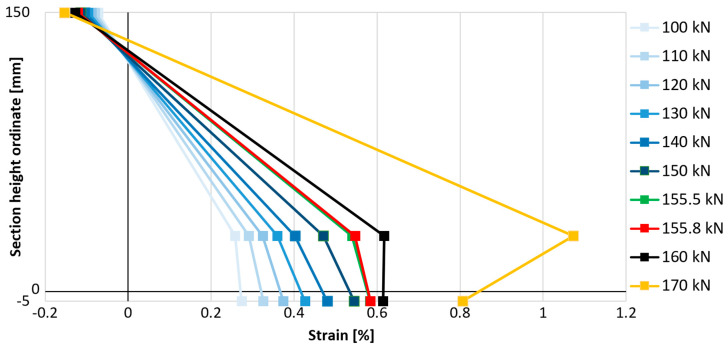
Distribution of longitudinal strains on the height of slab P_A_1.

**Figure 22:**
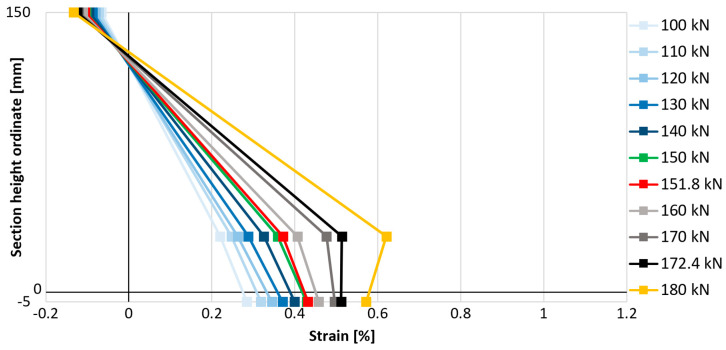
Distribution of longitudinal strains on the height of slab P_B_2.

**Figure 23:**
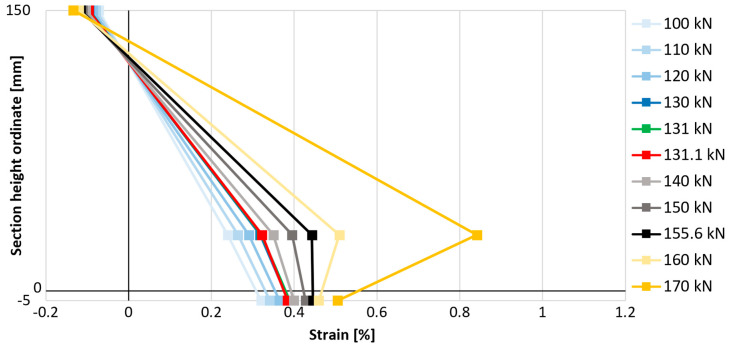
Distribution of longitudinal strains on the height of slab P_C_1.

In order to better understand the influence of anchorage on the effectiveness of the composite, it was decided to compare the strains on the compressed concrete surface, the strains of the tensile reinforcement, and the strains of the composite in the middle cross-section of the slab on one graph. This was intended to verify the moment of loss of proportionality of these strains associated with the loss of connection between the strengthened element and the composite. For this purpose, the strain gauge measurement results presented earlier were used and are compiled in Figure 21, Figure 22 and Figure 23. The value on the vertical axis of the graphs indicates the position of the measurement points on the height of the elements, where a value of 150 mm is the top concrete surface, a value of 30 mm is the axis of the main steel bottom reinforcement, a value of 0 mm is the bottom surface of concrete, and a value of −5 mm is the level of the strengthening. One representative example for each strengthened type of element is presented—with bar anchorage, cord anchorage, and without anchorage. The selection of representative examples was dictated primarily by the correct operation of all analyzed strain gauges through the most important phases of the elements’ performance.

The described moment of breaking the proportionality of the strain distribution in the cross-section is associated with a disproportionately large increase in longitudinal strains of reinforcement in relation to longitudinal strains of the composite. The reduction in the increases in strengthening strains is associated with the gradual loss of its adhesion to the concrete substrate, i.e., partial detachment of the composite. In each analyzed case, the strains of the composite increase after the moment of partial detachment, though not as dynamically as before the first signs of loss of adhesion of the composite. The point of breaking the proportionality of the strain distribution, hereinafter referred to as the strengthening detachment point, indicates the moment of initiation of strengthening failure. The load values associated with the detachment of the FRCM composite from the surface of the analyzed elements and their comparison with the value of the failure force of a given element are summarized in Table 7.

The detachment of strengthening in the element without mesh anchorage of the composite (P_C_1) began at a load of 131.1 kN, and in the case of strengthened elements with anchorage at a load of 155.8 kN for bar anchorage (P_A_1) and 151.8 kN for cord anchorage (P_B_2), which is a result 19% and 16% higher, indicating the effectiveness of these solutions in delaying the detachment of strengthening. When referring to the relative load level (in relation to the maximum force carried by the element), the most effective type of anchorage is also bar anchorage (type A), as detachment occurs at a load level equal to 74.2% of the maximum failure force, while in the element without anchorage, this value is 64.8%, and with cord anchorage (type B), 66.2%. When comparing these relative values, the increase in the effectiveness of strengthening due to its detachment is equal to 14% in the case of using bar anchorage (type A) and only 2% in the case of using cord anchorage (type B).

## 4. Discussion and Conclusions

In this research, we analyzed the influence of the effectiveness of the PBO-FRCM composite mesh anchorage, which was used to strengthen reinforced concrete slabs in flexure. Two types of PBO-FRCM mesh anchorage were proposed, which were applied separately in the composite strengthening of a reinforced concrete slab, and their behavior was compared with an element strengthened without anchorage and with an unstrengthened reinforced concrete element. The relationships described in this study indicate that the load–response behavior of a reinforced concrete slab element strengthened in flexure with one layer of PBO-FRCM composite (regardless of the presence and type of anchorage) and the control unstrengthened element is similar in the range of the first two deformation phases, up to the yielding of the main tensile reinforcement. PBO-FRCM strengthening in slab elements significantly increases the stiffness of the element only in the third phase of deformation after the reinforcement has yielded. The most important conclusions drawn from the described analysis are presented below.

- Applying a single layer of PBO-FRCM composite to strengthen the reinforced concrete slabs in flexure resulted in an 18–35% increase in bending load-bearing capacity. This strengthening technique simultaneously increased the load at reinforcement yielding by 17–30% and elevated the cracking threshold by 18–26%. Due to the relatively large differences in results between individual research series, the unambiguous influence of the composite mesh anchorage on these parameters was not established.
- Beyond enhancing the load-bearing capacity, the strengthening significantly improved element stiffness, particularly during the final phase of structural performance. Consequently, strengthened elements exhibited failure at substantially smaller deflections compared to control specimens, despite supporting higher failure loads, indicating a reduction in overall ductility.
- In the assessment of cracking, the increase in cracking force in elements strengthened with PBO-FRCM composites was confirmed. The further development of cracking in elements strengthened with PBO-FRCM composites is also limited in relation to the unstrengthened element, and the character of the element’s cracking is more favorable from the point of view of the behavior of the reinforced concrete element. However, no clear influence of anchorage on these properties was observed.
- The applied composite influenced the behavior of the strengthened elements, which became apparent through the reduction in strains of the main reinforcement bars and a significant reduction in the rate of increase in longitudinal strains of compressed concrete after yielding of the reinforcement.
- Measurements of the development of composite strains showed that the highest degree of utilization of the potential of PBO mesh in the applied FRCM composite, amounting to 47.4%, was achieved in the element in which bar anchorage of the composite mesh was applied (type A). Lower values of composite mesh utilization were recorded for cord anchorage (type B), on average, 31.3%, and when no mechanical anchorage of the composite mesh was applied (type C), on average, 30.2%.
- Mesh utilization analysis revealed that bar anchorage systems (type A) enabled the PBO mesh to reach 55% of its maximum potential strain capacity, significantly outperforming both cord anchorage (type B) and unanchored (type C) systems, which achieved only 42% and 43% utilization, respectively. It was, therefore, established that in this respect, cord anchorage is not effective (it does not increase the potential maximum strains of the mesh), and the application of bar anchorage of the mesh (type A) of the PBO FRCM composite caused a 28% increase in the mesh utilization indicator.
- The global comparison of strains for the entire element indicated that an important aspect that determines the failure of strengthening is the beginning of its detachment from the concrete surface, after which the strains do not increase proportionally to the strengthened element. Research has shown that the resistance of the PBO-FRCM composite to detachment is increased by 16% due to the application of cord anchorage (type B) and by as much as 19% due to the application of bar anchorage (type A). Additionally, based on the strain distribution, it was noticed that with further loading of the slab, even after detachment, the elements with bar anchorage of the composite mesh take the largest strains of the composite. The bar anchorage (type A), even after partial detachment of the strengthening, is able to continue carrying increasingly higher loads.

The PBO-FRCM composite is effective in flexural strengthening reinforced concrete slab elements, but premature failure does not allow for the full utilization of their potential. The application of mechanical anchorage of the mesh allows for increasing this effectiveness and better utilization of the high-strength fiber material. Based on the conducted research, a higher potential for increasing the effectiveness of strengthening is seen in bar anchorages (type A), which show the best performance and technological feasibility. The authors believe that the effectiveness of strengthening and the influence of different types of anchorage may have much greater significance in the case of the lower initial load-bearing capacity of the reinforced concrete slab; however, confirmation of this requires further research and analyses. In addition to mechanical anchorage of the composite mesh, possibilities to increase the effectiveness of PBO-FRCM strengthening should also be sought through modification of the adhesion zone between the fibers and the matrix or through modification of the matrix formula.

## Figures and Tables

**Table 1:** Summary of strength parameters of concrete and steel.

Parameter	Symbol	Value
Concrete compressive strength	*f*_c_	38.8 MPa
Concrete Young’s modulus	*E*_c_	35.2 GPa
8 mm rebars yielding stress	*f*_y,#8_	553 MPa
8 mm rebars ultimate stress	*f*_u,#8_	607 MPa
8 mm rebars Young’s modulus	*E*_s,#8_	197 GPa
10 mm rebars yielding stress	*f*_y,#10_	543 MPa
10 mm rebars ultimate stress	*f*_u,#10_	636 MPa
10 mm rebars Young’s modulus	*E*_s,#10_	187 GPa

**Table 2:** Summary of test elements.

Element	Strengthening	Anchorage
P_0	-	-
P_A_1	1 layer of PBO-FRCM	Bar anchorage (type A)
P_A_2		
P_B_1	1 layer of PBO-FRCM	Cord anchorage (type B)
P_B_2		
P_C_1	1 layer of PBO-FRCM	No anchorage (type C)
P_C_2		

**Table 3:** Summary of basic parameters related to individual phases of slab behavior.

Element	E_2_ [kN/mm]	ΔE_2_ [%]	E_3_ [kN/mm]	ΔE_3_ [%]	F_cr_ [kN]	ΔF_cr_ [%]	F_pl_ [kN]	ΔF_pl_ [%]	F_ult_ [kN]	ΔF_ult_ [%]
P_0	9.6	-	0.7	-	44.5	-	129.0	-	171.4	-
P_A_1	9.9	**+3%**	2.6	**+262%**	52.6	**+18%**	150.8	**+17%**	210.1	**+23%**
P_A_2	10.3	**+7%**	2.6	**+260%**	54.5	**+22%**	158.0	**+22%**	222.3	**+30%**
P_B_1	10.1	**+6%**	3.1	**+336%**	54.2	**+22%**	154.0	**+19%**	206.5	**+20%**
P_B_2	11.7	**+22%**	2.8	**+295%**	56.1	**+26%**	168.0	**+30%**	229.4	**+34%**
P_C_1	10.3	**+8%**	2.8	**+298%**	55.0	**+24%**	150.5	**+17%**	202.2	**+18%**
P_C_2	12.7	**+33%**	3.2	**+353%**	55.5	**+25%**	159.6	**+24%**	231.1	**+35%**

where **ΔX** is the increase in **X** value compared to P_0 element.

**Table 4:** Summary of key values related to slab cracking.

Element	*F*_cr,0.0_ [kN]	Δ*F*_cr,0.0_ [%]	*F*_cr,0.3_ [kN]	Δ*F*_cr,0.3_ [%]	Δ*F*_cr,0.4_ [kN]	*F*_cr,0.3_ [%]
P_0	35	-	68	-	89	-
P_A_1	53	**+51%**	110	**+62%**	120	**+35%**
P_A_2	56	**+60%**	108	**+59%**	119	**+34%**
P_B_1	56	**+60%**	95	**+40%**	130	**+46%**
P_B_2	57	**+63%**	97	**+43%**	130	**+46%**
P_C_1	57	**+63%**	102	**+50%**	122	**+37%**
P_C_2	55	**+57%**	105	**+54%**	118	**+33%**

where ***F*_cr,0.0_** is the load corresponding to the first visible cracking, ***F*_cr,0.3_** is the load corresponding to the crack width of 0.3 mm, ***F*_cr,0.4_** is the load corresponding to the crack width of 0.4 mm and **ΔX** is the increase in **X** value compared to P_0 element.

**Table 5:** Summary of loads associated with the breaking point of longitudinal strain curves for compressed concrete and the slopes of these curves after the breaking point.

Element	*F*_cc,pl_ [kN]	Δ*F*_cc,pl_ [%]	*slope*_cc,pl_ [kN/%_strain_]	Δ*slope*_cc,pl_ [%]
P_0	141.4	-	100.5	-
P_A_1	154.6	**+9%**	504.7	**+402%**
P_A_2	159.4	**+13%**	418.4	**+316%**
P_B_1	157.2	**+11%**	415.4	**+313%**
P_B_2	170.4	**+21%**	489.7	**+387%**
P_C_1	157.9	**+12%**	523.3	**+421%**
P_C_2	157.0	**+11%**	499.0	**+397%**

where ***F*_cc,pl_** is the load at which the compression concrete strain diagram changes its slope, ***slope*_cc,pl_** is the ratio between the load increment and compressed concrete strain increment in the final phase of the slab’s performance and **ΔX** is the increase in **X** value compared to P_0 element.

**Table 6:** Key parameters read from the graph of the relationship between FRCM composite strain and load for individual series and reference material data.

Element	*F*_frcm*,* pl_ [kN]	*ε*_frcm,max_ [%]	*ε*_frcm,ult_ [%]	*ε*_frcm,max/_*ε*_frcm,ult_ [-]	*F*_frcm,max_ [kN]	*F*_ult_ [kN]	*F*_frcm,max_/*F*_ult_ [-]	*I*_frcm,max_ [-]
P_A_1	71.0	1.02	2.15	**0.474**	179.7	210.1	0.855	**0.55**
P_B_1	74.7	0.59	2.15	**0.273**	136.8	206.5	0.663	**0.41**
P_B_2	67.5	0.76	2.15	**0.353**	194.4	229.4	0.847	**0.42**
P_C_1	56.3	0.84	2.15	**0.392**	201.6	202.2	0.997	**0.39**
P_C_2	55.6	0.46	2.15	**0.212**	104.5	231.1	0.452	**0.47**

where ***F*_frcm,pl_** is the load at which a sudden increase in FRCM reinforcement strain begins, ***F*_frcm,max_** is the load at which maximum composite strain was recorded, ***F*_ult_** is load on the test element causing its failure, ***ε*_frcm_**_,**max**_ is the maximum recorded strain of FRCM strengthening, ***ε*_frcm_**_,**ult**_ is the theoretical ultimate strain of FRCM composite causing its failure and ***I*_frcm,max_** is the mesh utilization indicator.

**Table 7:** Summary of loads associated with composite detachment.

Element	*F*_frcm,det_ [kN]	Δ*F*_frcm,det_ [%]	*F*_ult_ [kN]	*F*_frcm,det_/*F*_ult_ [-]	Δ*F*_frcm,det_/*F*_ult_ [%]
P_A_1	155.8	**+19%**	210.1	0.742	**+14%**
P_B_2	151.8	**+16%**	229.4	0.662	**+2%**
P_C_1	131.1	-	202.2	0.648	-

where ***F*_frcm,det_** is the load at which FRCM composite detaches from the strengthened element, ***F*_ult_** is the load on the test element causing its failure and **ΔX** is the increase in **X** value compared to P_0 element.

## Data Availability

The original contributions presented in this study are included in the article. Further inquiries can be directed to the corresponding author.
